# Effects of screw configuration on chemical properties and ginsenosides content of extruded ginseng

**DOI:** 10.1002/fsn3.1991

**Published:** 2020-11-21

**Authors:** Yu Zhang, Tie Jin, Gihyung Ryu, Yuxuan Gao

**Affiliations:** ^1^ Department of Food Science and Engineering Yanbian University Yanbian China; ^2^ Department of Food Science and Technology Kongju National University Yesan South Korea

**Keywords:** chemical properties, ginseng, ginsenosides, screw configuration, twin‐screw extrusion process

## Abstract

**Background:**

The purpose of this study was to investigate the effects of screw configuration on chemical properties and ginsenosides content of extruded ginseng and to select the most suitable screw configuration for the processing of ginseng.

**Method:**

The extrusion conditions were set as follows: moisture content (20%), barrel temperature (140°C), screw speed (200 rpm), and feeding rate (100 g/min).

**Result:**

The extruded ginseng of screw configuration 6 has the highest DPPH free radical scavenging rate, reducing power and total phenol, which is the most suitable configuration for the development of ginseng extract products. In addition, the extruded ginseng of screw configuration 9 has the highest content of total saponin, and the content of rare ginsenoside Rg3 which is scarcely present in the ginseng raw material powder was significantly increased. This intended that twin‐screw extrusion process enables the mutual conversion between ginsenosides and rare ginsenoside Rg3 had achieved.

**Conclusion:**

The extrusion process promotes the development and utilization of ginseng and provides theoretical basis for the design and development of screw configuration of twin‐screw extruded ginseng.

## Graphical abstract

The twin‐screw extruding function obviously improves the antioxidant ability of Ginseng extract. The content of total saponins in ginseng extract was increased by twin‐screw extruder. The content of rare ginsenoside Rg3 increased significantly after extrusion. Extensive additional research is necessary prior to industrial implementation.

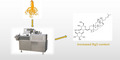

## 1 INTRODUCTION

Ginseng is a perennial herb of the Araliaceae family that has a high medicinal function (Lu et al., 2009). The main production places of ginseng are China, Korean Peninsula, Japan, and Russia (Baeg & So, 2013). Ginseng and its constituents have antitumor, antiaging, antibacterial, and immune‐regulating effects (Attele et al., 1999; Yu et al., 2019). It has been one of the most widely used herbs for thousands of years, and it is known as the “King of Grass” (Lu et al., (2009)). Ginsenosides is an important active ingredient in ginseng which has the effects of antitumor, anticancer, antioxidant, anti‐inflammatory, antifatigue, and antiradiation. According to the difference of its glycosidic ligand structure, it is divided into two types: damane tetracyclic triterpenoid saponins and oleanolic pentacyclic triterpenoid saponins. According to the differences of its glycosidic ligand groups, damacane ginsenosides are divided into ginsadiol type (mainly including Rb1, Rb2, Rc, Rd, Rg3) and ginsenoside type (mainly including Re, Rf, Rg1). Among them, ginsenoside Rg3 is basically not present in artificially cultivated ginsenoside and can usually be detected by appropriate transformation, which is a rare ginsenoside. Reply: This is the information about ginsenosides. Meanwhile, it can prevent hypertension, diabetes, and cardiovascular disease, enhance memory, and inhibit platelet activation and thrombosis in vivo (Wang, (2006); Moses et al., 2014; Kim et al., 2015; Quan et al., 2015; Christensen, 2009; Zhang et al., 2016; Vinh et al., 2017; Zhao et al., 2014; Lee et al., 2016; Jiang et al., 2014; Jiang et al., 2005; Wang et al., 2005).

Twin‐screw extrusion process is a combination of mixing, stirring, cutting, crushing, heating, cooking, sterilization, expansion, molding in one processing technology. Its biggest characteristic is high temperature, high pressure, and high shear force. In the extrusion process, the physical force acts on the raw material, so chemical changes occur. The cell wall of the material treated by twin‐screw extruder was destroyed, which contributed to the overflow of soluble components in the cells, and the extraction rate of soluble substances increased significantly. It can make some nutrient active substance structure change, better play the function of plant itself. The twin‐screw press technology has the advantages of wide material applicability, many kinds of products, simple production equipment, small area, low energy consumption, high production efficiency, and no pollution (Cheng & Friis, 2010; Wang et al., 2018). Screw is the core component of twin‐screw extruder, especially in transportation, shearing, mixing, compression, and so on. The arrangement and combination of different screw components is called screw configuration, and different screw configuration will affect system parameters and product parameters (Li, 2004; Ye et al., 2013; Zhang, 2010).

In recent years, some researchers have used twin‐screw extruders to process the ginseng that obviously improved the physical and chemical properties of the ginseng (Son & Ryu, 2009). Rg1, Rg2, and Rg3 were detected in extruded ginseng, and the content of total saponins was significantly increased (Ha et al., 2005). Ji et al. (2008) by using twin‐screw extrusion process found that when the moisture content of ginseng powder is 25% and the temperature of cylinder is 110°C, the extruded has high antioxidant activity. Gui et al. (2012) investigated the effect of twin‐screw extrusion process on the composition of ginseng as well. At present, the research of using twin‐screw extruder as biochemical reactor to process ginseng is based on barrel temperature, material water content, and screw speed, but the application of screw configuration in food industry is less.

Therefore, this study aimed at investigating the effects of screw configuration on the chemical properties and ginsenosides content of extruded ginseng, so that can provide theoretical basis for the design of screw configuration.

## 2 MATERIALS AND METHODS

### 2.1 Materials

The five‐year‐old cultivated ginseng was purchased in Shengxing planting cooperative in Ji'an City with a moisture content of 6.5%.

### 2.2 Design of screw configuration

**Figure 1:**
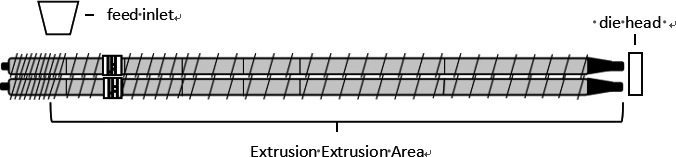
Schematic representation of the twin‐screw extruder barrel

**Figure 2:**
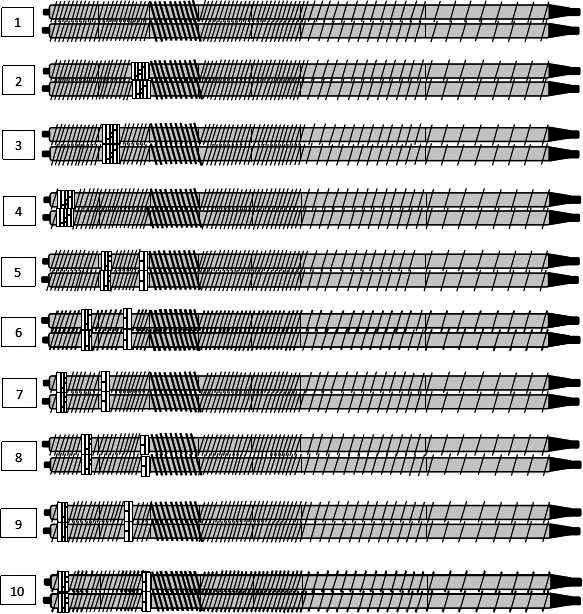
Schematic diagram of different screw configurations

**Figure 3:**
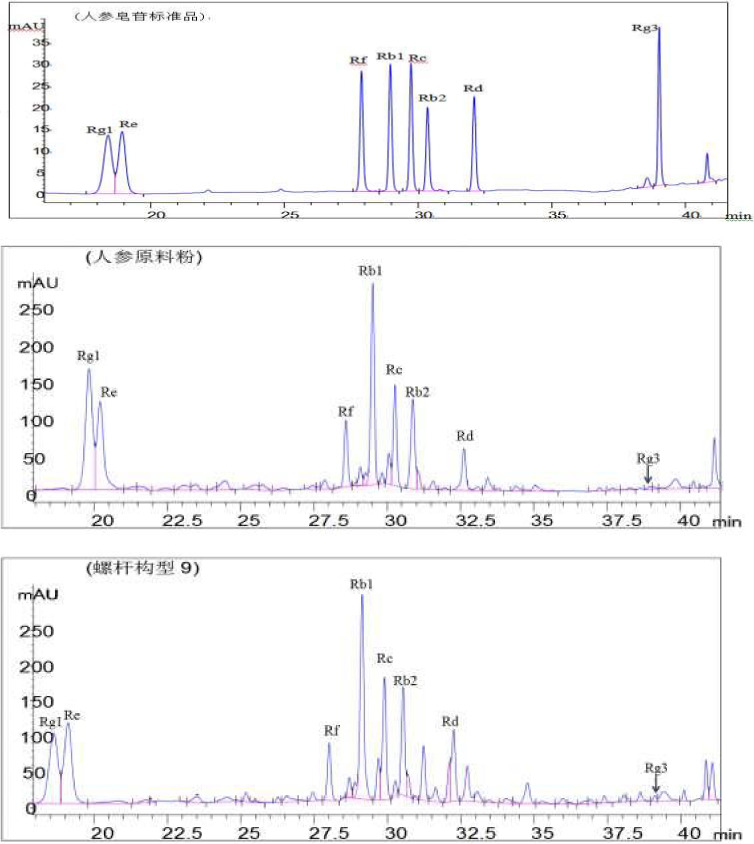
High‐performance liquid chromatography of standard and sample of ginsenosides

The screw of twin‐screw extruder can be divided into conveying section, shear melting section, and metering homogenization section from feeding end to extrusion end (Yang, 2013). Most of the reactions occur in the shear melting section and the melt transport section, so we focused on the screw configuration of these two parts. In this study, based on the previous research experiences and properly reducing the working quantity, the forward kneading element was used as the representative element to study the distance between the component and the die port (abbreviated as the part‐die distance) and the element spacing. Ten kinds of screw configurations with different component‐die distance and element spacing were designed. Taking different screw configurations as experimental factors, the effects of screw configuration on chemical properties and ginsenosides content of extruded ginseng were investigated. Ten kinds of screws with different configurations were designed in the test area of 15 cm at the die end, and the configuration of the nontest area remained unchanged. The screw combination from the feeding end was full pitch thread, 2/3 pitch thread, 1/2 screw pitch thread, and two sets of 1/2 screw pitch reverse thread elements with a total length of 6 cm were installed at 21 cm from the die port end. Part‐die distance design horizontal value is 0 cm, 6 cm, and 12 cm; element spacing design horizontal value is 0 cm, 6 cm, 9 cm, and 12 cm. Five forward kneading elements with a total thickness of 3 cm were used to replace the forward conveying elements in the fixed position in the 15 cm test area at the die port, respectively, which was used to study the effect of part‐die distance on the chemical properties of twin‐screw extruded ginseng. Five forward kneading elements were divided into two groups of 3 & 2 pieces to replace the forward conveying elements fixed in the 15 cm test area at the die port, which was used to study the effect of element spacing on the chemical properties of twin‐screw extruded ginseng. Configuration 1 was composed of all forward conveying elements in the experimental area and set as the control structure of all screw configurations. On the basis of configuration 1, configuration 2–4 were replaced by 5 forward kneading elements at 12 cm, 6 cm, and 0 cm from the die head, configuration 5–7 were replaced by 3 & 2 forward kneading elements at 6 & 12 cm, 3 & 9 cm, and 0 & 6 cm from the die head, configuration 8–9 were replaced with 3 & 2 forward kneading elements at 3 & 12 cm, 0 & 9 cm from the die head, and configuration 10 was replaced with 3&2 forward kneading elements at 0 & 12 cm from the die head (Figures 1, 2, 3).

### 2.3 Extrusion process

The ginseng powder (clean ginseng was dried at 50°C and crushed) was extruded with a corotating intermeshing twin‐screw extruder (Incheon Machinery Co.). The extrusion conditions were set as follows: ginseng powder water content was 20%, barrel temperature was 140°C, feed speed was 100 g/min, screw speed was 200 rpm, the diameter of the die head was 3.0 mm, the length of the screw was 742 mm, and the diameter of the screw was 32 mm. The ginseng extrusions were dried for 5 hr at 80°C. After drying, some samples were randomly selected to grind and pass 60mesh sieves for the determination of chemical properties and ginsenosides.

#### 2.3.1 Ginsenosides contents

0.50 g (dry weight) ginseng powder and 70 ml solution (MeOH: H_2_O = 2:1) were refluxed at 80°C for 3 h and repeated twice. The crude ginsenosides were obtained by rotary evaporating the leach liquor. Added 10 ml of distilled water and 20 ml of water saturated n‐butanol to the crude ginsenosides, the supernatant was taken after centrifugation. Added 30 ml of distilled water after extraction three times, the mixture was rotary evaporated at 60°C. The resulting solution was analyzed by HPLC after dissolution of methanol in the 3 ml chromatographic stage and filtration of the 0.45 μm membrane. Each sample was repeated three times to take the average value (Tong, 2018).

#### 2.3.2 Acidic polysaccharide contents

0.5 g ginseng powder and 40 ml distilled water were centrifugd after extracting at room temperature for 30 min. Take 1 ml of supernatant which diluted tenfold, and mixed it with 5 ml of sodium tetraborate/sulfuric acid. Add 100 μl of 1.5 mg/ml m‐hydroxybiphenyls to the mixed solution after 5 min of boiling water bath. Finally, the absorption value of the mixed solution at 520 nm was determined (Zhao, 2017).

#### 2.3.3 Total phenolic contents and antioxidant properties

1 g ginseng powder was extracted with 60 ml 80% ethanol by ultrasonic extraction at 60°C for 50 min. The mixture was centrifuged at 3000 rpm for 20 min. After centrifugation, the sediment was extracted the second time with 40 ml 80% ethanol. According to the method of Jin (2017), the total phenol content and DPPH radical scavenging activity were determined. And the reducing power was determined according to the method of Gui and Ryu (2014).

#### 2.3.4 Reducing sugar contents

5 g ginseng powder (dry weight) was extracted with 40 ml of distilled water for 5 min after heating at 80°C for 40 min. The supernatant was adjusted to 100 ml, and then, 1 ml of the diluted supernatant was mixed with 1.5 ml of dinitrosalicylic acid solution. The mixture was boiled at 100°C for 5 min. After heating, it was cooled in ice. The cooled solution was diluted to 25 ml using distilled water, and the absorbance was measured at 550 nm (Shu et al., 2010; Xue et al., 2015).

#### 2.3.5 Statistical analysis

Using SPSS 17.0 software to analyze the variance of the test results, Duncan's method was used for multiple comparisons, and the significant level was 0.05.

## 3 RESULTS AND ANALYSIS

### 3.1 Effect of screw configuration on the content of saponins in ginseng extrudates

The contents of 8 kinds of ginseng monomer saponins in each screw configuration were determined by HPLC. According to the Table 1, the contents of the terol type and diol type ginseng monomer saponins in ginseng powder have different degrees after being pressed by twin‐screw extrusion of ginseng powder, which indicates that the screw configuration has a significant influence on the content of ginseng monomer saponin. The results showed that the contents of triol ginsenoside Rg1 and Rf were the highest in ginseng raw materials, and the contents of diol ginsenoside Rb1, Rc, Rb2, Rd, Rg3 were the highest in configuration 9.

**Table 1:** Extrusion ginsenoside content of different screw configurations

	Saponins content (mg/g)								
	Rg1	Re	Rf	Rb1	Rc	Rb2	Rd	Rg3	Total amount of saponins
Raw material powder	2.84 ± 0.06^a^	4.29 ± 0.17^b^	1.27 ± 0.02^a^	8.24 ± 0.18^c^	1.98 ± 0.03^cd^	2.07 ± 0.06^b^	0.83 ± 0.01^e^	0.04 ± 0.03^g^	21.56 ± 0.46^b^
configuration 1	2.46 ± 0.06^b^	3.63 ± 0.01^de^	1.15 ± 0.00^e^	7.37 ± 0.03^f^	1.94 ± 0.01^d^	2.04 ± 0.01^b^	0.93 ± 0.14^cd^	0.11 ± 0.00^cd^	19.63 ± 0.11^d^
configuration 2	2.48 ± 0.05^b^	3.70 ± 0.10^d^	1.22 ± 0.03^cd^	8.43 ± 0.13 ^c^	2.01 ± 0.04^c^	1.74 ± 0.03^d^	1.12 ± 0.02^b^	0.09 ± 0.00^ef^	20.79 ± 0.38^c^
configuration 3	2.28 ± 0.03^e^	3.21 ± 0.04^g^	1.10 ± 0.01^fg^	7.04 ± 0.09^g^	1.59 ± 0.02^h^	1.36 ± 0.01^g^	0.74 ± 0.01^f^	0.09 ± 0.01^de^	17.42 ± 0.20^h^
configuration 4	2.25 ± 0.03^e^	2.66 ± 0.06^h^	1.08 ± 0.02^g^	6.76 ± 0.08^h^	1.43 ± 0.02^i^	1.21 ± 0.01^h^	0.72 ± 0.01^f^	0.07 ± 0.01^f^	16.19 ± 0.22^i^
configuration 5	2.42 ± 0.02^bc^	4.12 ± 0.04^c^	1.23 ± 0.01^bc^	8.83 ± 0.07^b^	2.26 ± 0.02^b^	1.86 ± 0.04^c^	0.97 ± 0.02^cd^	0.12 ± 0.00^bc^	21.80 ± 0.18^b^
configuration 6	2.25 ± 0.07^e^	3.37 ± 0.12^f^	1.19 ± 0.03^d^	7.81 ± 0.20^de^	1.75 ± 0.06^g^	1.50 ± 0.05^f^	0.99 ± 0.03^c^	0.08 ± 0.02^ef^	18.96 ± 0.57^ef^
configuration 7	2.36 ± 0.02^cd^	3.56 ± 0.03e	1.26 ± 0.01^ab^	7.87 ± 0.03^d^	1.86 ± 0.01^e^	1.61 ± 0.01^e^	0.71 ± 0.00^f^	0.10 ± 0.00^de^	19.31 ± 0.09^de^
configuration 8	2.29 ± 0.02^de^	3.20 ± 0.04^g^	1.13 ± 0.02^ef^	7.62 ± 0.06^e^	1.77 ± 0.01^fg^	1.55 ± 0.02^f^	0.91 ± 0.01^d^	0.09 ± 0.01^ef^	18.55 ± 0.18^fg^
configuration 9	2.28 ± 0.04^de^	5.04 ± 0.08^a^	1.22 ± 0.02^cd^	10.00 ± 0.13^a^	2.78 ± 0.04^a^	2.31 ± 0.02^a^	1.38 ± 0.01^a^	0.13 ± 0.00^ab^	25.16 ± 0.31^a^
configuration 10	2.23 ± 0.05^e^	3.23 ± 0.06^g^	1.10 ± 0.02^fg^	7.67 ± 0.10^de^	1.81 ± 0.03^f^	1.52 ± 0.02^f^	0.72 ± 0.01^f^	0.15 ± 0.01^a^	18.42 ± 0.25^g^

There was no significant difference in the data with the same letters in the same column (*p* > .05), but there was significant difference in the shoulder letters (*p* < .05).

Reply: Rg1 and Rf are triol ginsenosides.

It can be seen from Table 1 that the content of rare ginseng saponins Rg3, which almost does not exist in ginseng powder, increases significantly after extrusion, which indicates that the extrusion process can produce rare saponins unique to red ginseng. The results of this study are consistent with the conclusion of Kim et al. (Sung, 2013). When ginseng or red ginseng is extruded, some C‐20 hydroxyl groups may be isomerized when the C‐20 glycosyl residues can be cracked, and rare ginseng saponins Rg3 can be produced. The results of this study are close to those of Gui et al. (Zhang, 2014) in the study of the effect of extrusion cooking on the physical and chemical properties of white ginseng and red ginseng (powder). In ten different screw configurations, the content of Rg3 in ginseng extrudates with configuration 1 was significantly lower than that in configuration 9 and 10, which was significantly higher than that in configuration 2, 4, 6, and 8. But there was no significant difference in Rg3 content between configuration 1 and configuration 3, 5, and 7 (*p* > .05). This indicates that the forward kneading element has a significant effect on the content of rare saponins Rg3 in ginseng extrudates. When the element spacing was 0 cm, the Rg3 content in configuration 3 was significantly higher than that in configuration 4 (*p* < .05), but there was no significant difference between configuration 3 and configuration 2 (*p* > .05). When the element spacing was 6 cm, the Rg3 content in configuration 6 was significantly higher than that in configuration 5 (*p* < .05), and when the element spacing was 9 cm, the content of Rg3 in configuration 9 was significantly higher than that in configuration 8 (*p* < .05). These show that the component‐die distance of the forward kneading element has a significant effect on the content of rare ginsenosides Rg3 in ginseng extrudates. When the distance between the part and the module is 0 cm, and the distance between component and die increased gradually, the content of Rg3 in configuration 9 and 10 was significantly higher than that in configuration 4 and 7 (*p* < .05). It indicates that the element spacing of forward kneading element has a significant effect on the content of rare ginsenosides Rg3 in ginseng extrudates. Among the ten different screw configurations, the content of rare ginsenosides Rg3 in ginseng extrudates with configuration 9 is the highest. In conclusion, the component‐die distance and element spacing of forward kneading elements have significant effects on the content of rare ginsenosides Rg3 in twin‐screw extrusion of ginseng.

From Table 1, the content of total saponins in extruded ginseng powder changed significantly after extrusion (*p* < .05). In ten different screw configurations, the content of total saponins in configuration 1 was significantly lower than that in configuration 2, 5, and 9 (*p* < .05), which was significantly higher than that in configuration 3, 4, 6, 8, and 10 (*p* < .05). But there was no significant difference between configuration 1 and configuration 7 (*p* > .05). This indicates that the positive kneading element has a significant effect on the content of total saponins in ginseng extrudates. When the distance between elements was 0 cm, the content of total saponins in configuration 2 was significantly higher than that in configuration 3 (*p* < .05). When the element spacing was 6 cm, the content of total saponins in configuration 5 was significantly higher than that in configuration 6, and 7. And when the element spacing was 9 cm, the content of total saponins in configuration 8 was significantly lower than that in configuration 9 (*p* < .05). These results show that the component‐mold distance of the forward kneading element has a significant effect on the total saponins content of ginseng extrudates. When the distance between the part and the module is 0 cm, and the distance between component and die increased gradually, the content of total saponins in configuration 9, 7, 10, and 4 had significant difference, and the content of total saponins decreased in turn. This indicates that the distance between the elements of the forward kneading element has a significant effect on the content of total saponins in Panax ginseng extrudates. Among the ten different screw configurations, the total saponins content of ginseng extrudates in configuration 9 was the highest. In summary, the component‐die distance and element spacing of forward kneading elements have significant effects on the content of total saponins in twin‐screw extrusion of Panax ginseng.

### 3.2 Effect of screw configuration on the content of acid polysaccharide in extruded ginseng

It can be seen from Table 2 that the content of acid polysaccharide in ginseng powder increased significantly after extrusion (*p* < .05). The results of this study are consistent with the conclusion of Gui et al. (Zhang, 2014), that the content of acid polysaccharide increases and the content of reducing sugar decreases significantly after twin‐screw extrusion of Panax ginseng powder. The results of this study are consistent with the conclusion of Ma et al. (2019) that the content of acid polysaccharide in ginseng powder increased significantly after extrusion. Ha and Ryu (2005) concluded that the increase of acid polysaccharide content may be due to the high temperature, high pressure, and high shear force of ginseng powder during extrusion, which leads to the destruction of cell wall and the release of sugar. In ten different kinds of screw configurations, the content of acidic polysaccharide in configuration 1 was significantly lower than that in configuration 2, 3, and 4 (*p* < .05), but there was no significant difference between configuration 1 and other configurations (*p* > .05). This indicates that the forward kneading element has a significant effect on the acid polysaccharide content of ginseng extrudates. When the distance between elements was 0 cm, the content of acidic polysaccharide in configuration 3 was significantly higher than that in configuration 4 (*p* < .05), but there was no significant difference between configuration 2 and configuration 3. And When the distance between the elements was 6 cm, there was no significant difference in the content of acidic polysaccharides in configuration 5, 6, and 7 (*p* > .05). When the distance between the elements was 9 cm, there was no significant difference in the content of acidic polysaccharides in configuration 8 and 9 (*p* > .05). It indicates that the component‐mold distance of forward kneading element has a significant effect on the content of acid polysaccharide in ginseng extrudate. When the distance between the part and the module is 0 cm, and the distance between component and die increased gradually, there is no significant difference in the content of acidic polysaccharide in configuration 4, 7, 9, and 10, which indicates that the element spacing of forward kneading element has no significant effect on the content of acidic polysaccharide in ginseng extrudate. Among the configurations, the acid polysaccharide content of ginseng extrudates in configuration 3 was the highest. In conclusion, the component‐die distance and element spacing of the forward kneading element have significant effect on the acid polysaccharide content of ginseng extrudates, but the element spacing has no significant effect on the acid polysaccharide content of ginseng extrudates.

**Table 2:**
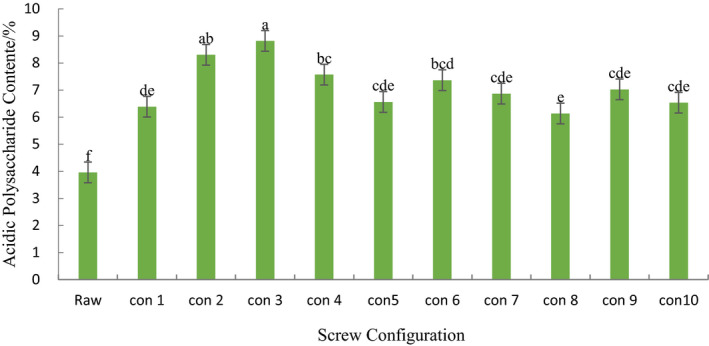
Acidic polysaccharie content of extruded ginseng on different screw configurations

### 3.3 Effect of screw configuration on the content of total phenols in extruded ginseng

It can be seen from Table 2 that the total phenol content of ginseng extrudates was significantly increased (*p* < .05). This is consistent with the conclusion that Ryu (2006) obtained a significant increase in the total phenol content of ginseng after twin‐screw extrusion. In ten different screw configurations, the total phenol content of screw configuration 1 was significantly lower than that of configuration 3,5,6,7 (*p* < .05), but there was no significant difference with the rest of the configurations. This indicates that the positive kneading element affects the total phenol content of ginseng extrusion. When the distance between elements was 0 cm, the total phenol content in configuration 3 was significantly higher than that in configuration 2, 4 (*p* < .05). When the distance between elements was 6 cm, the content of total phenols in configuration 6 was significantly higher than that in configuration 5 (*p* < .05), but there was no significant difference between configuration 6 and configuration 7. When the distance between elements was 9 cm, there was no significant difference in the total phenol content of ginseng extrudates in configuration 8 and 9 (*p* > .05). This shows that the component‐mold distance of the forward kneading element has a significant effect on the total phenol content of ginseng extrudates. When the distance between the part and the module is 0 cm, and the distance between component and die increased gradually, the content of total phenols in configuration 7 was significantly higher than that in configuration 4, 9, and 10, which indicated that the element spacing of forward kneading elements had a significant effect on the total phenol content of ginseng extrudates. In ten kinds of screw, the total phenols of ginseng extrudates with configuration 6 are the highest. In conclusion, the component‐die distance and element spacing of the forward kneading element have significant effects on the total phenol content of extruded ginseng.

### 3.4 Effect of Screw configuration on DPPH Free radical scavenging rate of extruded ginseng

It can be seen from Table 2 that the DPPH free radical scavenging rate of ginseng extrudates increased significantly after extrusion (*p* < .05). This shows that the Maillard reaction of ginseng powder after extruded by screw improves the oxidation resistance of ginseng extrudates. In ten different screw configurations, the scavenging rate of DPPH radical in screw configuration with forward kneading element was significantly higher than that in control screw (*p* < .05). It indicates that the positive kneading element has a significant effect on the scavenging rate of DPPH radical in ginseng extrudate. When the distance between elements was 0 cm, the scavenging rate of DPPH radical in configuration 3 was significantly higher than that in configuration 2, 4 (*p* < .05). When the distance between elements was 6 cm, the scavenging rate of DPPH radical in configuration 6 and 7 were significantly higher than that in configuration 5 (*p* < .05). When the distance between elements was 9 cm, the scavenging rate of DPPH radical in configuration 9 was significantly higher than that in configuration 8 (*p* < .05). This shows that the component‐die distance of the forward kneading element has a significant effect on the DPPH free radical scavenging rate of ginseng extrudates. When the distance between the part and the module is 0 cm, and the distance between component and die increased gradually, the scavenging rate of DPPH radical in configuration 7 was significantly higher than that in configuration 4, 9, and 10. This indicates that the distance between the elements of the forward kneading element has a significant effect on the DPPH free radical scavenging rate of ginseng extrudates. Among the ten kinds of screw, the DPPH radical scavenging rate of ginseng extruder with configuration 6 was the highest and the antioxidant capacity was the strongest. In conclusion, the component‐die distance and element spacing of forward kneading elements have significant effects on the scavenging rate of DPPH free radicals in extruded ginseng.

### 3.5 Effect of Screw configuration on reduction Force of ginseng extrudates

It can be seen from Table 2 that the reduction ability of ginseng powder increased significantly after extrusion (*p* < .05). Among the ten different screw configurations, the reduction force of the screw configuration with forward kneading element was significantly higher than that in the control screw (*p* < .05), which indicated that the positive kneading element increased the pressure and shear force between materials, and Maillard reaction increased the melanin‐like substance and led to the increase of reduction force. When the element spacing was 0 cm, the reduction force of screw configuration 3 was significantly higher than that of configuration 2, 4. When the distance between elements was 6 cm, the reduction force of configuration 6 was significantly higher than that of configuration 5 (*p* < .05). When the distance between elements was 9 cm, the reduction force of configuration 8 was significantly lower than that of configuration 9 (*p* < .05). This shows that the part‐die distance of the forward kneading element has a significant effect on the reduction force of ginseng extrudates. When the distance between the part and the die is 0 cm, and the element spacing increases gradually, there were significant differences in reducing power of ginseng extrudates between configuration 7, 9, 10, and 4 (*p* < .05), and the reduction force decreased in turn. This shows that the element spacing of the forward kneading element has a significant effect on the reduction force of the ginseng extrusion material. Among the ten kinds of screw, the reduction force of ginseng extrudates with configuration 6 is the highest. To summarize, the component‐die distance and element spacing of the forward kneading element have a significant effect on the reduction force of extruded ginseng.

### 3.6 Effect of screw configuration on the content of reducing sugar in extruded ginseng

As can be seen from Table 2, the content of reducing sugar of the ginseng extrusion decreased significantly after the ginseng powder was extrudated (*p* < .05). The reason that the content of reducing sugar of the ginseng extrudates is reduced may be that the Maillard reaction of the material consumes a lot of reducing sugar. Fang et al. (2016) concluded that the reduction of the content of reducing sugar in the process of extrusion was mainly the effect of Maillard reaction. Gui et al. (2012) founded that he reason for the significant decrease in the content of reducing sugar after the ginseng is pressed is due to the Maillard reaction with the amino acid during the high temperature. Suzuki et al. (2004) found that red ginseng was subjected to high temperature, high pressure, and high shear force during processing, and a large amount of reducing sugar reacted with amino acids in Maillard. In the ten different screw configurations, the reducing sugar content of the configuration 1 was significantly lower than that of the configurations 2, 3, 4, 5, 6, 7 (*p* < .05), but significantly higher than the configuration 8, 9, 10 (*p* < .05). This indicates that the positive kneading element has a significant effect on the reducing sugar content of the ginseng extrudate. When the distance between elements was 0 cm, the content of reducing sugar in configuration 4 was significantly higher than that in configuration 2 and 3 (*p* < .05). When the distance between elements was 6 cm, the content of reducing sugar in configuration 6 was significantly higher than that in configuration 5, 7 (*p* < .05). When the distance between elements was 9 cm, the content of reducing sugar in configuration 8 was significantly higher than that in configuration 9(*p* < .05). This indicates that the component‐to‐mold distance of the forward kneading element has a significant effect on the reducing sugar of the ginseng extrudates. When the distance between the part and the module is 0 cm, and the distance between component and die increased gradually, there was a significant difference between the reducing sugar contents of the configurations 4,7,10, and 9 (*p* < .05), and the content was decreased in this order. This indicates that the element spacing of the forward kneading element has a significant effect on the reducing sugar content of the ginseng extrudate. In the ten screws, the sugar content of the ginseng extrudate of the configuration 6 was the highest. Generally speaking, the component‐die distance and element spacing of the forward kneading element have significant effects on the reducing sugar content of extruded ginseng (Tables 3, 4, 5, 6).

**Table 3:**
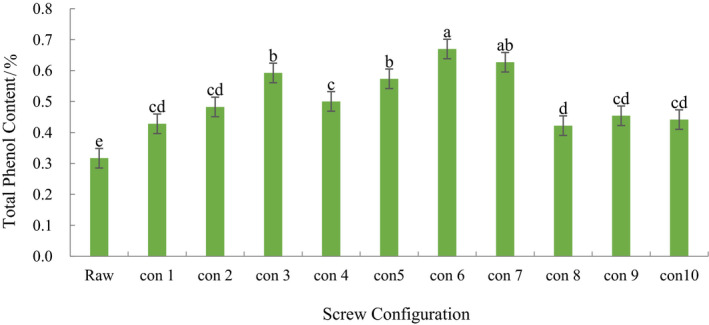
Total phenol content of extruded ginseng on different screw configurations

**Table 4:**
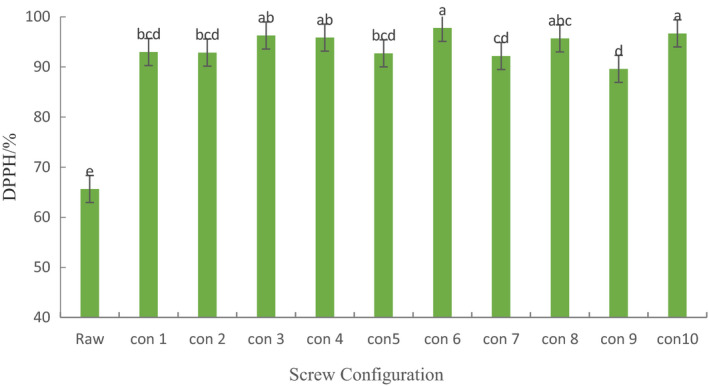
DPPH radical scavenging rate of extruded ginseng on different screw configurations

**Table 5:**
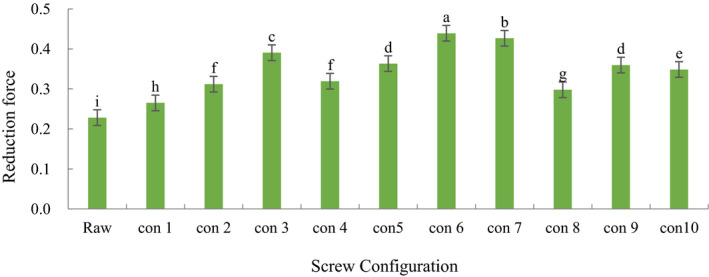
Reduction force of extruded ginseng on different screw configurations

**Table 6:**
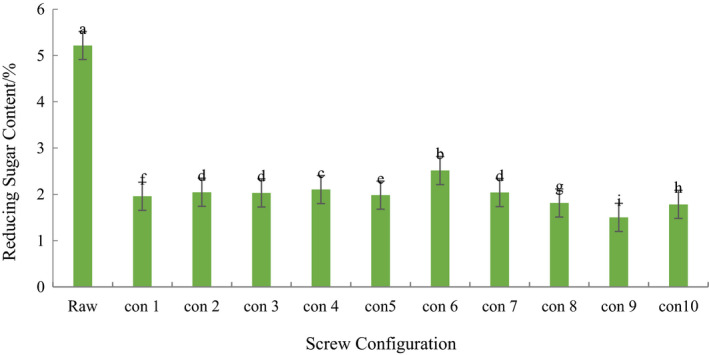
Reducing sugar content of extruded ginseng on different screw configurations

## 4 CONCLUSION

The antioxidant capacity of ginseng is one of the important characteristics of ginseng. After the ginseng powder was extruded by different configurations of screw, the oxidation resistance of ginseng extrudates was significantly increased. Among them, the scavenging rate of DPPH radical and the reduction force of ginseng extrudates in configuration 6 were the highest, so the antioxidant capacity was the strongest. The content of ginsenoside is also one of the important indexes to measure the quality of ginseng. After the ginseng raw material powder was extruded by screw configuration 9, the content of total saponins in ginseng extrudates was the highest, which was 14.31% higher than that before extrusion. And the content of rare ginsenosides Rg3, which hardly existed in ginseng raw material powder, was significantly increased by 73.33%.

## Data Availability

The data that support the findings of this study are available from the corresponding author upon reasonable request.
